# The Function of Adsorption, Photo-Oxidation, and Humic Acid Using Air Backwashing in Integrated Water Treatment of Multichannel Ceramic MF and PP Particles

**DOI:** 10.3390/membranes10020028

**Published:** 2020-02-11

**Authors:** Sangwoo Park, Dongyeop Kim, Jin Yong Park

**Affiliations:** Department of Environmental Sciences & Biotechnology, Hallym University, Chunchon 24252, Korea; psw2216@naver.com (S.P.); kmdomyp@naver.com (D.K.)

**Keywords:** air backwashing, microfiltration, photo-oxidation, adsorption, polypropylene particles, integrated system, water treatment, ceramic

## Abstract

For advanced water treatment, function of microfiltration (MF), adsorption, photo-oxidation, humic acid (HA), and polypropylene (PP) particles on membrane fouling and decay effectiveness were investigated in an integrated water treatment, of multichannel ceramic MF and PP particles, using UV radiation and air backwashing. The synthetic feed was organized with HA and kaolin. The membrane fouling resistance (R_f_) of the (MF + PP) system presented the lowermost, and amplified intensely from the (MF + UV) to MF system. The percentages of MF and adsorption by PP particles for turbidity treatment were 87.6% and 3.8%, individually; however, the percentages of MF and adsorption by PP particles for dissolved organic matters (DOM) treatment were 27.9% and 5.0%, respectively. The decay effectiveness of turbidity presented the greatest 95.4% at HA of 10 mg/L; however, that of DOM increased as HA concentration ascended. The ultimate R_f_ after 180 min procedure showed the maximum at 30 g/L of PP particles concentration, and improved dramatically, as PP particles decreased. Finally, the maximum V_T_ was acquired at 30 and 50 g/L of PP particles, because flux preserved greater throughout the procedure. The decay effectiveness of turbidity and DOM showed the maximal 95.4% and 56.8% at 40 and 50 g/L of PP particles, respectively.

## 1. Introduction

The adsorption or precipitation of organic and inorganic components at membrane surface produces a membrane fouling and a permeate flux reduction, that decreases membrane life and increases the cleaning costs of a membrane. Technologies for governing membrane fouling, that is the main problem in the profitable application of a membrane filtration system, remain inadequate, although significant advancement has been completed at membrane fouling [1,2]. Biofouling is a serious concern in water and wastewater treatment by a membrane system, as it significantly negotiates the effectiveness of the treatment systems. It is problematic to regulate, and important financial properties have been devoted to the improvement of efficient biofouling checking and governing schemes [3].

A main fouling component of low-pressure membrane separation is a natural organic matter (NOM). Numerous defensive methods for inhibiting NOM fouling have been established and broadly verified, for instance, activated carbon adsorption, ion exchange, coagulation, oxidation, and adsorption [4]. A new adsorbent, such as heated aluminum oxide powder, was utilized in a completely automated pilot water treatment process to eliminate NOM in the surface water [5]. In this research, periodic air backwashing was achieved for reducing the membrane fouling.

In recent times, the integrated technique of membrane filtration and photo-oxidation by UV radiation has successfully resolved the aforementioned membrane fouling issues [6]. The integrated technique not only preserves the benefits of each technique, but also creates synergistic influences that overwhelm the bounds of an individual technique of membrane filtration and photo-oxidation. Furthermore, contaminants, for instance NOM, can be oxidized by UV radiation, and organic matters are decayed to some extent via governing the retention period in the reaction process. Additionally, the membrane is a selective barrier to filtrate matters. In conclusion, the integrated technique improves photo-oxidation effectiveness and attains an outstanding treated water quality. The influence of UV-radiation on the nano-integrated polyether sulfone-NanoZnO membrane has been investigated by Kusworo and Utomo [7] in relation to flux and treatment effectiveness. Furthermore, an evaluation of the effectiveness of surface water treatment in integrated procedures, by joining numerous advanced oxidation methods and ultrafiltration (UF), was reported by Szymański et al. [8]. In this research, an integrated system of alumina membrane and polypropylene (PP) particles using UV radiation could be utilized as tertiary water treatment.

The ceramic membranes, applied in this research, have various benefits, that are high chemical, thermal, and mechanical resistance, in addition to an extensive lifetime. Those are frugally reasonable as paralleled with polymeric membranes, owing to a practically enduring lifetime [9]. At the present time, the ceramic membranes have been extensively utilized in water/wastewater treatment all over the world [10,11]. The fouling effect of soluble algal organic materials (AOMs) has been inspected using a multichannel ceramic microfiltration (MF) membrane by [12]. The interface effect of aquatic humic materials and AOM on ceramic MF membrane fouling has been researched by [13]. The ceramic MF membranes were developed in the tube form based on natural crystalline silicon dioxide by [14]. The active absorption phase of a composite ceramic membrane and their adsorption characteristics were reported by [15].

Photo-oxidation has the properties of extraordinary effectiveness, little energy depletion, and an extensively various utilization, by mineralizing organic materials to trivial inorganic molecules and oxidizing most of them, specifically biological nondegradable organic pollutants. Moreover, it is an outstanding technique in water treatment. Therefore, the photo-oxidation technique utilized in this research has been utilized generally [16,17,18,19,20,21]. Furthermore, degradation of HA, that was a component of the synthetic feed applied in this research, by means of the photoelectrocatalysis (PEC) system, was examined by [22].

In our water treatment team, the influence of operational situations in an integrated system of numerous inorganic membranes and titanium dioxide (TiO_2_) photocatalyst-coated PP particles were reported in “Desalination and Water Treatment” [23] and “Membr. J.” [24]. Moreover, the effects of adsorption and photo-oxidation in an integrated system of tubular carbon fiber UF and PP particles using UV radiation and water backwashing were published in “Desalination and Water Treatment” [25].

In this research, the influence of microfiltration (MF), adsorption, photo-oxidation, DOM, and pure PP particles on membrane fouling and decay effectiveness was investigated in an integrated water treatment system, of multichannel ceramic MF and PP particles, using UV radiation and air backwashing. This study included the distinctive utilization of pure PP particles to inspect the influence of MF, adsorption, photo-oxidation, DOM, and PP particles in the integrated system. The periodic air backwashing was accomplished during 10 s per 10 min separation to decline membrane fouling. The PP particles were fluidized in the space between the module case and the ceramic membrane, to eliminate the DOM and turbid materials. The results of DOM and PP particles effects in this study were compared with the previous work by [26] in the integrated water treatment system, of multichannel ceramic MF and PP particles, using water backwashing.

## 2. Materials and Methods 

The seven-channel ceramic MF membrane (HC04) utilized in the research was supplied from Dongseo Industry (Seoul, Korea), and its pore sizes were 0.4 μm. The membrane manufacturing company produced two kinds of pore alumina membrane, of 0.4 and 1.0 μm, and the HC04 membrane (0.4 μm) was selected due to the smaller pore size. A specification of the HC04 alumina membrane, which was manufactured with α-alumina coating on α-alumina support, is summarized in Table 1. The size of polypropylene (PP) particles utilized in this research, was 4–6 mm, and the average weight of the PP particles was 39.9 mg. As a synthetic water in this study, an amount of HA and kaolin was melted in purified water, in place of NOMs and suspended inorganic materials of natural water basis. For the photo-oxidation of DOM, 352 nm ultraviolet (UV) light was exposed from the acryl module outside via two UV lamps (F8T5BLB, Sankyo, Tokyo, Japan).

The PP particles were fluidized between the module inside and the alumina membrane outside, to eliminate the DOM and turbid materials in the integrated module. A 100-mesh (0.150 mm) sieve, which was tremendously tinier than 4–6 mm of the PP particles applied in this research, was mounted in the permeated flow side of the integrated module outlet, to avoid the PP particles leaking with the treated flux. 

**Figure 1:**
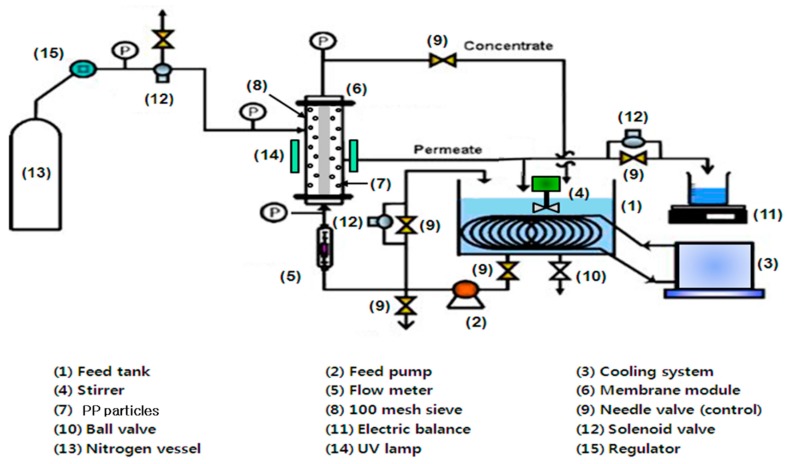
Apparatus of the integrated water treatment system, of tubular multichannel alumina ceramic microfiltration and pure polypropylene particles, using periodic air backwashing and UV radiation.

The advanced water treatment system, that used in our previous research [27] for paper wastewater treatment using only the multichannel membrane, was applied in this study utilizing an integrated module of multichannel alumina MF and pure PP particles with UV radiation, as displayed in Figure 1. Air backwashing was periodically performed from the outside to the inside of the tubular multichannel alumina ceramic membrane by solenoid valves and timer. The integrated module was occupied up with 40 g/L of the pure PP particles, in the space of the ceramic membrane outside and the acryl module case inside, and the PP particles were fluidized in the space. The feed container was occupied with 10 L of the synthetic water created by HA and kaolin, and the feed water temperature was continually preserved at 20 °C by utilizing a persistent temperature circulator (Model 1146, VWR, Atlanta, GA, USA). Furthermore, the synthetic feed water was uninterruptedly mixed by an agitator, to preserve the uniform feed, and it flowed to the multichannel alumina membrane inside, and its flow rate was preserved at 1.0 L/min using a pump (Procon, Standex Co., Smyna, TN, USA). The feed flow rate was gaged by a flow meter (NP-127, Tokyo Keiso, Tokyo, Japan). The pressure in the integrated module was continuously preserved via regulating both valves of a feed bypass and a concentrate tube. The feed water was filtered by the multichannel alumina membrane, and contacted the PP particles. The treated water was gaged by an electric balance (Ohaus, Newark, NJ, USA). The treated water was returned to the feed container after measuring the mass, without only 5 ml for water quality analysis. Additionally, the water quality of feed tank was checked at every sampling time, to maintain the feed concentration. The treated and the concentrate water circulated to the feed container to sustain a persistent feed concentration throughout the integrated system procedure. A physical cleaning was achieved in the multichannel membrane inside by a brush after each experiment, and the treated flux was gaged to determine the resistances of irreversible and reversible membrane fouling.

At first, only a MF system without pure PP particles and UV radiation, a (MF + UV) system using UV radiation, and a (MF + PP) system using the PP particles were individually performed at 6 mg/L of HA, which was in the mid concentration of the HA effect range. In addition, those were paralleled with the integrated (MF + PP + UV) system of MF and PP particles using UV radiation to investigate percentages of the DOM/turbidity decay efficiencies by multichannel alumina MF, photo-oxidation, and adsorption. The PP bead concentration was 40 g/L, which was the minimum irreversible membrane fouling (R_if_) condition of the previous research using water backwashing [26]. The filtration time (FT) and air backwashing time (BT) were held at 10 min and 10 s, correspondingly, to diminish the membrane fouling. As a second experiment, HA was altered in the range of 2–10 mg/L in the synthetic water, to examine the DOM influence on the membrane fouling and decay efficiencies; however, kaolin was held at 30 mg/L. As the third experiment, the PP particles concentration was altered in the range of 0–50 g/L at the space of the membrane outside and module inside, to inspect the PP particles influence on the decay effectiveness and membrane fouling. 

The treated flux (J) was gaged, and the membrane fouling resistance (R_f_) was computed during 180 min of total operation time at each investigational situation. The trans-membrane pressure (TMP) was preserved continuously at 0.8 bar, and the air backwashing pressure at 1.0 bar. Moreover, the feed water temperature was maintained at 20 °C and the feed flow rate at 1.0 L/min in every investigation. 

The feed and treated water quality was analyzed six times at each experiment to evaluate the decay efficiencies of DOM and turbid matters. UV_254_ absorbance was measured via a UV spectrophotometer (Genesys 10 UV, Thermo, Pittsburgh, PA, USA) to analyze DOM. Furthermore, turbidity was checked via a turbidimeter (2100N, Hach, Ames, IA, USA) to measure turbid matters. The detection bounds of UV spectrophotometer and turbidimeter were −0.1–3.0 cm^−1^ (±0.001 cm^−1^) and 0–4000 NTU (±0.001 NTU), correspondingly. The UV_254_ absorbance, representing DOM, was measured after filtering turbid matters, which was blocking materials for the UV_254_ absorbance, using a 0.2 μm syringe filter. 

After running each experimental study, all of the synthetic water was liquidated from the integrated system, and purified water was passed continuously through all of the line for 15 min to wash the alumina membrane and device. After the cleaning procedure, the pure PP particles and the membrane were detached from the system, and the alumina membrane was incinerated at 550 °C in a furnace, to combust fouling components in the membrane inside for the period of 30 min. After keeping the membrane at room temperature for one day, it was submerged in a mess cylinder of 15% nitric acid (HNO_3_) for the period of one day, and in a 0.25 N sodium hydroxide (NaOH) solution for the period of 180 min, to wash out organic or inorganic pollutants, and then held in purified water for one day to clean. Before operating a new investigation, the permeated flux of purified water (J_w_) was gaged for inspecting the membrane recovery of 95% in regular operation. The recovered membrane, the flux difference of which was below 5%, was utilized in all of the experiments to diminish a membrane situation effect on the decay effectiveness.

## 3. Results and Discussions

The function of MF, adsorption, photo-oxidation, DOM, and PP particles on membrane fouling and decay effectiveness were inspected in an integrated water treatment system, of multichannel ceramic MF and PP particles, with UV radiation and air backwashing. Permeate flux (J) data were used to compute resistances of the membrane, boundary layer, and membrane fouling (R_m_, R_b_, R_f_) by utilizing the resistance-in-series filtration model (J = ΔP / (R_m_ + R_b_ + R_f_)), as the equivalent procedure to former research [28], where ΔP means the transmembrane pressure. The model was shortened to J = ΔP / R_m_, for the reason that the resistances of boundary layer and membrane fouling did not exist for a fresh membrane. The R_m_ could be computed by utilizing permeate flux data for a fresh membrane. The recovered membrane, the flux difference of which was below 5%, was applied in all of the investigations to diminish the membrane situation effect on the decay effectiveness. Actually, the membrane resistance (R_m_) could be calculated from the flux of the recovered membrane, which was not a real fresh membrane, because of the membrane fouling included. At initial time of the synthetic feed, the model was reformed to J_0_ = ΔP / (R_m_ + R_b_), and R_b_ could be computed by using J_0_ and R_m_ values, where J_0_ is the initial permeate flux. After physical cleaning by a brush in the membrane channel inside, the resistances of irreversible and reversible membrane fouling (R_if_, R_rf_) could be determined from the permeate flux values (J).

### 3.1. The Function of Photo-Oxidation, Adsorption, and Membrane Filtration

**Figure 2:**
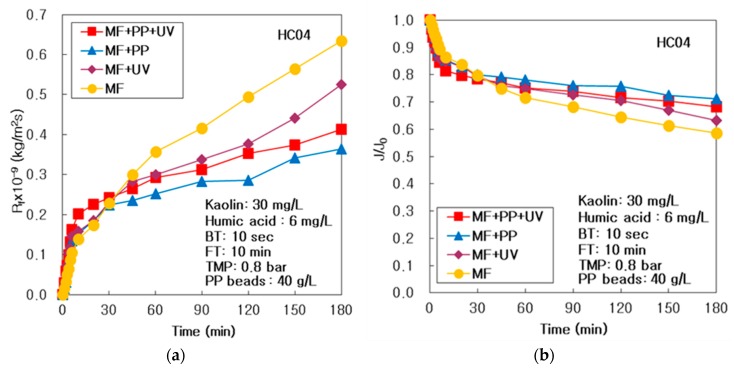
Function of microfiltration, adsorption of PP particles, and photo-oxidation of UV radiation in the integrated system of multichannel ceramic MF and PP particles using periodic air backwashing: (**a**) Membrane fouling resistance and (**b**) dimensionless treated flux.

The (MF + PP) system using pure PP particles devoid of UV radiation, (MF + UV) system using UV radiation, and simply the MF system devoid of any PP particles and UV radiation were accomplished individually at HA 6 mg/L, and matched with the (MF + PP + UV) system of MF and PP particles with UV light. The membrane fouling resistance (R_f_) of (MF + PP + UV), (MF + PP), (MF + UV), and only MF systems at 6 mg/L of HA were matched in Figure 2a for the duration of 3 h. The R_f_ of the (MF + PP) system presented the lowest, and amplified intensely from the (MF + UV) to the MF system; however, that of the (MF + PP + UV) system was not the lowest value. It proved that the membrane fouling could be diminished powerfully by pure PP particles adsorption and the UV radiation photo-oxidation in this integrated water treatment system. 

As arranged in Table 2, the membrane resistance (R_m_) was regulated at persistent value by incinerating the membrane in a furnace, and cleaning it in acidic and alkali solution. After 180 min procedure, a final R_f_ (R_f,180_) was the maximum at 0.635 × 10^9^ kg/m^2^/s in the MF system, because of the highest R_b_, that was created by a concentration polarization on the membrane surface; however, the R_f,180_ was the lowest at 0.364 × 10^9^ kg/m^2^/s in the (MF + PP) system. The R_rf_ improved intensely when the system was made simpler, from the (MF + PP + UV) to the MF system in Table 2; however, the R_if_ was the minimal at 0.225 × 10^9^ kg/m^2^/s in the (MF + PP) system and the maximal at 0.351 × 10^9^ kg/m^2^/s in the MF system.

As shown in Figure 2b, to examine a comparative deterioration of treated flux, the dimensionless treated flux (J/J_0_), where J_0_ is the initial treated flux which was predicted by extrapolation using the first two J values at 1 and 2 min, could preserve the maximal in the (MF + PP) system and the minimal in the MF system. It proved that the J/J_0_ in (MF + PP) could be greater than those of only the MF and the (MF + UV) systems, for the reason that pure PP particles adsorption could decrease the membrane fouling more powerfully than the UV radiation photo-oxidation. As summarized in Table 2, the final value of J/J_0_ (J_180_/J_0_) was the maximal at 0.711 in the (MF + PP) system after 180 min operation, which was 1.22 times greater than the minimal of 0.585 in the single MF system. The highest value of 224 L/m^2^hr of permeate flux (J) could be attained in the (MF + PP) system, for the reason that the membrane fouling was repressed further powerfully via pure PP particles adsorption in the (MF + PP) system than in the single MF or (MF + UV) systems. Finally, the total permeate volume (V_T_) was the maximal at 15.3 L in the (MF + PP) system and the minimal at 13.9 L in the MF system during 180 min operation.

As paralleled in Table 3 and Table 4, the decay efficiencies of turbidity and DOM, that can be examined by UV_254_ absorbance, reduced clearly when making the system simpler, step by step, from (MF + PP + UV), (MF + PP), and (MF + UV) to MF with air backwashing. The percentages of decay effectiveness by MF, adsorption, and UV photo-oxidation in this integrated system using air backwashing could be computed by deducting the turbid or organic materials decay effectiveness of MF, from those of (MF + PP) and (MF + UV) systems correspondingly, as prepared in Table 5. The percentage of decay effectiveness by photo-oxidation with PP particles could be obtained by deducting the DOM or turbidity decay effectiveness of (MF + PP) from that of the (MF + PP + UV) system.

The percentages of MF, the PP particles adsorption, the UV radiation photo-oxidation, and the photo-oxidation with PP particles for turbidity decay were 87.6%, 3.8%, 3.5%, and 3.6%, respectively, as shown Table 5. It verified that the function of MF and adsorption was important; however, those of UV photo-oxidation with/without the pure PP particles did not perform a dominant function to reduce the suspended inorganic matters in this integrated system, because inorganic materials, such as kaolin, could not be decayed by UV photo-oxidation. 

However, the percentages of MF, PP particles adsorption, UV photo-oxidation, and photo-oxidation with PP particles for DOM (UV_254_ absorbance) decay were 27.9%, 5.0%, 4.4%, and 0.2%, respectively, as shown in Table 5. It proved that the function of MF was the most dominant, and those of adsorption by pure PP particles and UV photo-oxidation were more significant than that of photo-oxidation with PP particles for the DOM reduction in the integrated water treatment system. The adsorption on non-porous PP particles could not be effective, but pollutants could be coated and adsorbed on the PP particles surface. The pure PP particles adsorption and UV photo-oxidation could forcefully diminish membrane fouling, for the reason that the photo-oxidation and adsorption forced the main part of DOM decomposition in this integrated system using air backwashing.

### 3.2. The Influence of Humic Acid (HA) on Decay Effectiveness and Membrane Fouling

**Figure 3:**
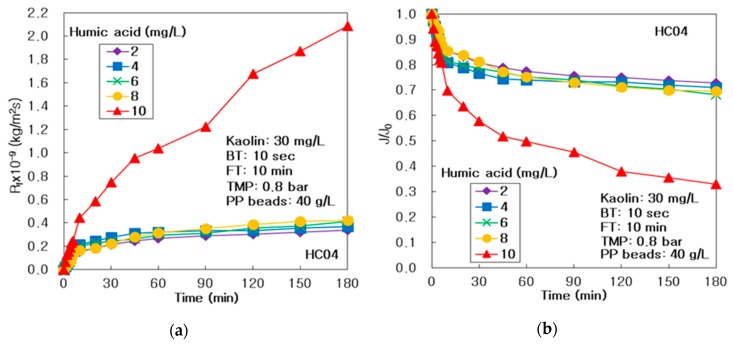
Influence of humic acid in the integrated system, of multichannel ceramic MF and pure PP particles, using UV radiation and periodic air backwashing: (**a**) Membrane fouling resistance and (**b**) dimensionless treated flux.

As compared in Figure 3a, the resistances of membrane fouling (R_f_) improved a small amount, as HA concentration was increased from 2 to 8 mg/L; however, R_f_ at HA of 10 mg/L was maintained at very high values during the 180 min operation. It proved that the dissolved organic materials, like as humic acid, could generate more rigorously the membrane fouling on the surface and inside of alumina membrane, beyond the specific HA concentration, such as 8 mg/L, in the integrated system of membrane and PP particles. 

In the former research by [26] about the integrated system of the similar multichannel MF (HC10, pore size 1.0 μm) and the equivalent pure PP particles using water backwashing, the R_f_ was highly influenced by HA and nearly persistent at 2–6 mg/L of HA; however, it improved intensely, as HA concentration was increased from 6 to 8 mg/L, and then diminished abruptly from 8 to 10 mg/L of HA. It proved that DOM, such as HA, could motivate the membrane fouling further strictly on the alumina membrane surface and inside; nevertheless, the dense fouling cake on the membrane might be detached via water backwashing at HA of 10 mg/L.

The feed flowrate (Q) was 1.0 L/min (1.667 × 10^−5^ m^3^/s) into the alumina membrane channel. The cross sectional area (A) of the membrane was 8.796 × 10^−5^ m^2^ (A = (π/4)D^2^ × 7, where D = 0.004 m). The mean linear velocity (v) inside the membrane channel was 0.1895 m/s (v = Q/A). The Reynolds number (Re) was 755.2 at 20 °C water, which had a laminar flow lower than 2100 (Re = ρvD/μ, where ρ = 998.23 kg/m^3^, μ = 1.002 × 10^−3^ kg/m/s at 20 °C water). The turn-over number (N) of the feed was 18 (N = t/T, where operation time t = 180 min, feed flow time T = V/Q = (10 L)/(1.0 L/min) = 10 min).

As arranged in Table 6, the R_f,180_ after 3 h procedure at 10 mg/L of humic acid was the highest 2.087 × 10^9^ kg/m^2^/s, which was 6.17 times greater than the lowest R_f,180_ of 0.338 × 10^9^ kg/m^2^/s at 2 mg/L; however, the values increased slowly from 0.338 × 10^9^ to 0.421 × 10^9^ kg/m^2^/s when HA was amplified from 2 to 8 mg/L. It proved that the sever membrane fouling could occur beyond the specific HA concentration in this integrated system of ceramic membrane and PP particles using UV radiation. In addition, the R_rf,180_ increased slowly from 0.078 × 10^9^ to 0.152 × 10^9^ kg/m^2^/s as HA was amplified from 2 to 8 mg/L, and increased dramatically to 0.864 × 10^9^ kg/m^2^/s at HA of 10 mg/L. Additionally, the R_if,180_ increased suddenly from 0.269 × 10^9^ to 1.223 × 10^9^ kg/m^2^/s as HA was increased from 8 to 10 mg/L.

In the former research by [26] about the integrated system using water backwashing, as compared in Table 6, the membrane resistance (R_m_) was regulated persistently by the furnace incineration and acid/alkali cleaning. After 180 min procedure, the final R_f_ (R_f,180_) at HA of 8 mg/L was 1.963 × 10^9^ kg/m^2^/s, which was 1.76 times higher than 1.113 × 10^9^ kg/m^2^/s of the R_f,180_ value at HA of 6 mg/L. The R_f_ value (1.246 × 10^9^ kg/m^2^/s) with water backwashing was much lower than 2.087 × 10^9^ kg/m^2^/s of air backwashing at HA of 10 mg/L. It proved that water backwashing might be more efficient to inhibit the membrane fouling at high HA condition, than air backwashing, in this integrated system of ceramic membrane and PP particles.

As compared in Figure 3b, the J/J_0_ showed a little reduction trend HA concentration was increased from 2 to 8 mg/L; however, it decreased abruptly at HA of 10 mg/L, for the reason that the membrane fouling might be produced via additional organic macromolecules beyond the specific HA concentration. As presented in Table 6, after 180 min operation the final J_180_/J_0_ dropped a little from 0.728 to 0.695 as HA was increased from to 8 mg/L; however, it diminished dramatically to 0.329 at 10 mg/L, due to severe membrane fouling beyond HA of 8 mg/L.

In the former research by [26] about the integrated system using water backwashing, as compared in Table 6, the final J/J_0_ (J_180_/J_0_) after 180 min procedure was 0.437 at HA of 6 mg/L, which was 1.33 times greater than 0.307 at HA of 8 mg/L. It proved that the permeated flux could showed a different trend, depending on backwashing media of air or water. The J_180_/J_0_ value using air backwashing was 0.329, which was much lower than 0.406 of water backwashing at HA of 10 mg/L. It verified that water backwashing was able to maintain the higher flux more effectively than air backwashing in this integrated system of ceramic membrane and PP particles.

Furthermore, the V_T_ decreased slowly from 15.21 L at HA 2 mg/L to 13.93 L at 8 mg/L; however, it suddenly dropped to the lowest, 8.18 L, at HA of 10 mg/L, as displayed in Table 6. It proved that NOM concentration, like HA, could be a major cause of membrane fouling in this integrated water treatment system of multichannel alumina membrane and pure PP particles using air backwashing.

In the former research by [26], about the integrated system using water backwashing, as compared in Table 6, the V_T_ of 12.48 L in HA of 4 mg/L presented 1.38 times more than 9.05 L of V_T_ in HA of 8 mg/L. It proved that the HA effect on the total treated volume might be more severe for air backwashing than water backwashing.

As paralleled in Table 7, the decay effectiveness of turbidity improved a small amount, from 94.7% to 95.4%, as HA concentration was increased. It proved that the suspended particles, like kaolin, might be rejected more successfully at higher HA concentrations, due to the secondary membrane, which was the gel deposit on the membrane surface, via the robust membrane fouling in the integrated system of ceramic membrane and PP particles.

In the former study by [26], about the integrated system using water backwashing, as arranged in Table 7, the turbidity decay effectiveness displayed a growing trend in relation to increasing HA concentration. This phenomenon for water backwashing showed a similar trend to the results of this study with air backwashing. It proved that the backwashing media could not disturb the decay effectiveness of turbidity in the integrated system of PP particles and multichannel alumina membrane.

In the former research by [26], for the integrated system using water backwashing, as shown in paralleled data in Table 8, the DOM decay effectiveness improved as HA concentration was increased, and in conclusion presented the extreme result of 49.7% in HA of 10 mg/L. It proved that DOM might be treated more excellently in an extreme DOM situation in this integrated system of multichannel ceramic MF and PP particles using water backwashing. The DOM decay effectiveness for air backwashing was higher below HA of 8 mg/L; nevertheless, it showed lower in HA of 10 mg/L than that for water backwashing. It proved that the air backwashing was more effective for DOM decay than the water backwashing in this integrated system.

As presented in Table 8, the decay effectiveness of DOM showed just about persistent values in the range of 33.1% and 34.6%, while HA amplified from 2 to 6 mg/L; however, it increased to 40.9% at 8 mg/L, and displayed the maximum of 42.5% at 10 mg/L. The treated water quality of DOM improved a little faster than the feed water quality, when HA changed from 2 to 6 mg/L, for the reason that HA might not be attached enough via PP particles and decomposed by UV photo-oxidation. On the other hand, the treated water quality of DOM enlarged more slowly than the feed water quality beyond HA 6 mg/L, since organic materials could be rejected by the secondary membrane formed on the membrane surface via sturdy membrane fouling.

### 3.3. The Influence of Pure PP Particles on Decay Effectiveness and Membrane Fouling 

**Figure 4:**
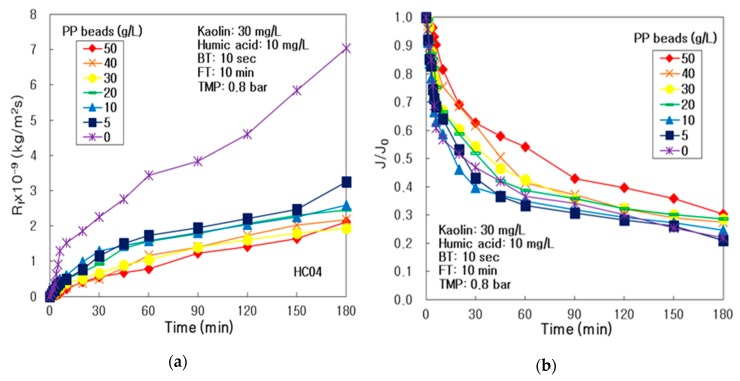
Influence of pure PP particles concentration in the integrated system, of multichannel ceramic MF and PP particles, using UV radiation and periodic air backwashing: (**a**) Membrane fouling resistance and (**b**) dimensionless treated flux.

As compared in Figure 4a, the R_f_ presented the highest at 0 g/L of pure PP particles, and the lowest at 50 g/L during 180 min in this integrated system of multichannel ceramic membrane and PP particles using air backwashing. It means that the more amount of PP particles could inhibit more tremendously the membrane fouling in the integrated water treatment system.

As arranged in Table 9, the R_f,180_ after 3 h operation presented the maximum (i.e., 7.045 × 10^9^ kg/m^2^/s) at 0 g/L of PP particles, which was 3.65 times more than 1.932 × 10^9^ kg/m^2^/s of the R_f,180_ at 30 g/L of PP particles. The R_rf_ was the lowest, 0.353 × 10^9^ kg/m^2^/s, at 30 g/L, and the maximal, 3.654 × 10^9^ kg/m^2^/s, at 0 g/L of PP particles; nevertheless, the R_if_ showed the minimal, 1.144 × 10^9^ kg/m^2^/s, at 50 g/L and the maximal, 3.390 × 10^9^ kg/m^2^/s, at 0 g/L of PP particles. The R_b_ presented the lowest at 30 g/L and the highest at 0 g/L. It proved that 30 g/L of PP particles might be the optimal condition in the integrated system, due to the lowest reversible membrane fouling and concentration polarization; however, the irreversible membrane fouling might be prevented at 50 g/L of PP particles, which was the highest quantity of PP particles in our experimental range. The concentration polarization at the membrane surface could be checked by the resistance of the boundary layer (R_b_).

In former research via [26], about the integrated system using water backwashing, as compared in Table 9, the R_f,180_ after 3 h was the maximal, 12.94 × 10^9^ kg/m^2^/s, at 50 g/L, which was 3.06 times more than 4.23 × 10^9^ kg/m^2^/s at 0 g/L of PP particles. The R_rf_ presented a growing tendency when PP particles concentration was increased from 0 to 50 g/L; on the other hand, the least R_if_ was at 40 g/L and the extreme was at 0 g/L of PP particles. The results were in agreement with the research results using air backwashing. It means that the backwashing media did not affect the membrane fouling in the integrated system of multichannel ceramic membrane and PP particles.

As compared in Figure 4b, the J/J_0_ presented the peak at 50 g/L of PP particles for the duration of the 3 h process, and the least in the range of 0–10 g/L in the integrated system. As arranged in Table 9, the J_0_ and J_180_ presented the extreme at 30 g/L of PP particles, and J_180_ amplified, with the increasing of PP particles concentration; nevertheless, after the 3 h process, the J_180_/J_0_ on the PP particles of 50 g/L was the maximal, 0.304, due to the least membrane fouling. In conclusion, the V_T_ was the best, 2.09 L, for PP particles of 30 and 50 g/L, for the reason that J was preserved greater during the 3 h process on PP particles of 30 g/L. It means that the best condition of PP particles might be 30 g/L, due to the highest J_180_/J_0_ and V_T_.

In the former research by [26], for the integrated system using water backwashing, as paralleled in Table 9, the J_0_ and J_180_ presented a reducing tendency, from 0 to 40 g/L and 50 g/L, of the PP particles, respectively. This phenomenon came from the reason that the R_b_ and R_f_ displayed an increasing trend from 0 to 40 g/L and to 50 g/L of the PP particles, correspondingly. As a final point, after the 3 h procedure, the J_180_/J_0_ on the PP particles of 0 g/L presented the extreme result, 0.230, which was 2.13 times more than 0.108 at 50 g/L. On the other hand, the V_T_ displayed the maximum 11.75 L at the PP particles of 5 g/L. It came from the reason that J was preserved greater throughout the procedure than those of other PP particles situations. It proved that the backwashing media could affect the treated flux in the integrated system of multichannel ceramic membrane and PP particles.

As prepared in Table 10, the turbidity decay effectiveness showed an increasing tendency, while increasing the PP particles concentration; however, it presented the maximal 95.7% on the PP particles at 50 g/L. It proved that the ideal PP particles concentration to treat the turbid components, such as kaolin, could be 50 g/L in the integrated system, because the PP particles adsorption was the most effective on the PP particles at 50 g/L.

In the former study by [26], about the integrated system using water backwashing, as paralleled in Table 10, the turbidity decay efficiencies presented nearly consistent results at the scope of 97.5% and 98.9%, unrestricted by the PP particles concentration. It proved that the turbid components might be rejected excellently, unrestricted by PP particles concentration in the integrated water treatment system. It verified that the backwashing media could effect the decay efficiencies of turbidity, because the result with water backwashing did not agree with this study with air backwashing.

As paralleled in Table 11, the DOM decay effectiveness did not show a special trend as the PP particles concentration was amplified; however, it was the best, 56.8%, at 40 g/L of PP particles and dropped to the lowest, 37.8%, at 50 g/L. It verified that the DOM could be treated the most excellently on the PP particles of 40 g/L, because UV for photo-oxidation could transfer enough to the module inside on the PP particles of 40 g/L. Finally, the optimal condition could be 40 g/L for treating turbid matter and DOM in the integrated system of multichannel alumina membrane and PP particles.

In the former research via [26], about the integrated system using water backwashing, as paralleled in Table 11, the DOM decay effectiveness did not display a tendency; on the other hand, it presented the best, 51.3%, on the PP particles of 5 g/L. It proved that the ideal PP particles concentration could be 5 g/L to decay DOM in the integrated water treatment system using water backwashing. In addition, it verified that the backwashing media could best affect the PP particles concentration for the DOM decay in the integrated system.

This integrated system could be applied as a tertiary process in water or wastewater treatment. A total 6.60 g of PP particles was supplied for the PP particles of 50 g/L in this integrated system of 1.0 L/min (60 L/h), because the volume between the ceramic membrane outside and the module inside was 0.165 L. For water treatment of 1 m^3^/h, PP particles of 110 g would be used, if this integrated system of 60 L/h was scaled up to a 16.7 times (= (1000 L/h)/(60 L/h)) practical system.

## 4. Conclusions

In this research, the function of MF, photo-oxidation, adsorption, DOM, and PP particles on decay effectiveness and membrane fouling were examined in the integrated water treatment system, of multichannel ceramic MF and PP particles, using UV radiation and air backwashing. This integrated system could be utilized for paper wastewater treatment, because it includes a high suspended solid concentration, organic compounds, and some strong chemicals, such as acid. In conclusion, these investigations could extract out the following results:

1. The membrane fouling resistance of the (MF + PP) system presented the highest, and improved intensely from the (MF + UV) to the MF system; however, that of the (MF + PP + UV) system was not the lowest value. It verified that pure PP particles adsorption and UV radiation photo-oxidation could diminish the membrane fouling powerfully in the integrated system.
2. The percentages of MF, the pure PP particles adsorption, the UV radiation photo-oxidation, and the photo-oxidation using the PP particles for turbidity decay displayed 87.6%, 3.8%, 3.5%, and 3.6%, respectively. It proved that the function of MF and adsorption was important; however, those of UV photo-oxidation with/without pure PP particles were not dominant. However, the percentages of MF, PP particles adsorption, and UV photo-oxidation with/without PP particles were 27.9%, 5.0%, 4.4%, and 0.2% for DOM decay, correspondingly. It proved that the function of MF was the most dominant, and those of PP particles adsorption and UV photo-oxidation without PP particles were more important than that of photo-oxidation using PP particles, for the DOM decay.
3. The membrane fouling resistance improved intensely, as HA concentration was increased from 8 to 10 mg/L. Finally, the maximum V_T_ could be attained at HA of 2 mg/L. The turbidity decay effectiveness displayed the highest 95.4% on HA of 10 mg/L; however, the DOM decay effectiveness increased with increasing HA concentration. This verified that DOM might show more principal function than turbid matter for membrane fouling.
4. After the 3 h process, the final membrane fouling resistance displayed the maximum at the PP particles of 30 g/L, and improved dramatically as PP particles were decreased. Finally, the V_T_ was attained at the PP particles of 30 and 50 g/L, because the treated flux preserved greater throughout the procedure.
5. The turbidity and DOM decay effectiveness exhibited the highest, 95.7% and 56.8%, at the PP particles of 50 and 40 g/L, respectively. It means that the ideal PP particles concentration could be 40 –50 g/L for turbidity and DOM decay, because the PP particles adsorption was the most effective and UV for photo-oxidation could transfer enough to the module inside at the PP particles of 40–50 g/L.

## Figures and Tables

**Table 1:** Specification of the multichannel alumina microfiltration (MF) membrane utilized here.

Model Name	HC04
Membrane pore size (μm)	0.4
Number of channels	7
Outer diameter (mm)	20
Inner diameter (mm)	4
Total length (mm)	241
Membrane surface area (cm^2^)	212
Membrane material	α-alumina
Manufacturing company	Dongseo Industry

**Table 2:** Function of microfiltration, PP particles adsorption, and UV radiation photo-oxidation on separation elements in the integrated system, of multichannel ceramic MF and pure PP particles, using periodic air backwashing at HA 6 mg/L.

System	MF + PP + UV	MF + PP	MF + UV	MF
R_m_ × 10^−9^ (kg/m^2^/s)	0.888	0.893	0.898	0.887
R_b_ × 10^−9^ (kg/m^2^/s)	0.001	0.005	0.001	0.009
R_f,180_ × 10^−9^ (kg/m^2^/s)	0.413	0.364	0.526	0.635
R_if_ × 10^−9^ (kg/m^2^/s)	0.320	0.225	0.319	0.351
R_rf_ × 10^−9^ (kg/m^2^/s)	0.093	0.140	0.207	0.284
J_0_ (L/m^2^/hr)	318	314	314	315
J_180_ (L/m^2^/hr)	217	224	198	184
J_180_/J_0_	0.683	0.711	0.631	0.585
V_T_ (L)	14.9	15.3	14.5	13.9

**Table 3:** Decay effectiveness of turbidity for the function of microfiltration, adsorption, and photo-oxidation at humic acid 6 mg/L in the integrated system, of multichannel ceramic MF and pure PP particles, using air backwashing.

System	Turbidity (NTU)				Average Decay Effectiveness (%)
	Feed Water		Treated Water		
	Range	Average	Range	Average	
MF + PP + UV	21.7–30.9	25.8	1.09–1.28	1.17	95.0
MF + PP	26.8–27.6	27.2	2.09–2.54	2.35	91.4
MF + UV	28.2–31.4	30.0	2.57–2.74	2.66	91.1
MF	27.3–31.5	29.6	2.59–3.94	3.68	87.6

**Table 4:** Decay effectiveness of DOM (UV_254_ absorbance) for the function of microfiltration, adsorption, and photo-oxidation at HA 6 mg/L in the integrated system, of multichannel ceramic MF and pure PP particles, using air backwashing.

System	UV_254_ Absorbance (cm^−1^)				Average Decay Effectiveness (%)
	Feed Water		Treated Water		
	Range	Average	Range	Average	
MF + PP + UV	0.157–0.222	0.184	0.093–0.159	0.123	33.1
MF + PP	0.199–0.226	0.210	0.126–0.165	0.141	32.9
MF + UV	0.227–0.287	0.258	0.159–0.191	0.175	32.3
MF	0.235–0.267	0.249	0.165–0.201	0.179	27.9

**Table 5:** Decay effectiveness percentages of microfiltration, adsorption, and photo-oxidation in the integrated system, of multichannel ceramic MF and pure PP particles, using air backwashing at 6 mg/L of humic acid.

Percentage of Decay Effectiveness	Turbidity	DOM
Microfiltration (%)	87.6	27.9
Adsorption (%) = (MF + PP) − MF	3.8	5.0
UV photo-oxidation (%) = (MF + UF) − MF	3.5	4.4
Photo-oxidation with PP particles (%) = (MF + PP + UV) − (MF + PP)	3.6	0.2
Total decay effectiveness (%)	95.0	33.1

**Table 6:** Influence of humic acid on separation elements in the integrated water treatment system, of multichannel ceramic MF and pure PP particles, using air backwashing (FT 10 min, BT 10 s); paralleled with outcomes of water backwashing.

Backwashing	HA (mg/L)	2	4	6	8	10
Air	R_m_ × 10^−9^ (kg/m^2^/s)	0.896	0.901	0.888	0.923	0.924
	R_b_ × 10^−9^ (kg/m^2^/s)	0.008	0.008	0.001	0.036	0.101
	R_f,180_ × 10^−9^ (kg/m^2^/s)	0.338	0.371	0.413	0.421	2.087
	R_if_ × 10^−9^ (kg/m^2^/s)	0.260	0.282	0.320	0.269	1.223
	R_rf_ × 10^−9^ (kg/m^2^/s)	0.078	0.089	0.093	0.152	0.864
	J_0_ (L/m^2^/hr)	312	311	318	294	275
	J_180_ (L/m^2^/hr)	227	221	217	205	91
	J_180_/J_0_	0.728	0.710	0.683	0.695	0.329
	V_T_ (L)	15.21	14.61	14.93	13.93	8.18
Water [26]	R_m_ × 10^−9^ (kg/m^2^/s)	0.840	0.803	0.864	0.831	0.798
	R_b_ × 10^−9^ (kg/m^2^/s)	0.072	0.030	0.001	0.038	0.053
	R_f,180_ × 10^−9^ (kg/m^2^/s)	1.165	1.137	1.113	1.963	1.246
	R_if_ × 10^−9^ (kg/m^2^/s)	0.044	0.228	0.408	0.453	1.037
	R_rf_ × 10^−9^ (kg/m^2^/s)	1.121	0.909	0.705	1.510	0.209
	J_0_ (L/m^2^/hr)	310	339	326	325	332
	J_180_ (L/m^2^/hr)	136	143	143	100	135
	J_180_/J_0_	0.439	0.423	0.437	0.307	0.406
	V_T_ (L)	10.6	12.48	11.30	9.05	9.39

**Table 7:** Decay effectiveness of turbidity for effect of humic acid in the integrated system, of multichannel ceramic MF and pure PP particles, using air backwashing (FT 10 min, BT 10 s); paralleled with outcomes of water backwashing.

Kaolin (mg/L)	HA (mg/L)	Turbidity (NTU)				Average Decay Effectiveness (%)	
		Feed Water		Treated Water		Backwashing Media	
		Range	Average	Range	Average	Air	Water [26]
30	2	18.7–27.8	22.3	1.10–1.22	1.18	94.7	97.1
	4	20.3–28.5	24.8	1.12–1.34	1.26	94.9	98.1
	6	21.7–30.9	25.8	1.09–1.28	1.17	95.0	98.3
	8	22.6–27.6	24.9	1.11–1.23	1.16	95.3	98.7
	10	24.9–34.2	29.0	0.354–2.94	1.32	95.4	98.4

**Table 8:** Decay effectiveness of dissolved organic matter (UV_254_ absorbance) for effect of humic acid in the integrated system, of multichannel ceramic MF and pure PP particles, using air backwashing (FT 10 min, BT 10 s); paralleled with consequences of water backwashing.

Kaolin (mg/L)	HA (mg/L)	UV_254_ Absorbance (cm^−1^)				Average Decay Effectiveness (%)	
		Feed Water		Treated Water		Backwashing Media	
		Range	Average	Range	Average	Air	Water [26]
30	2	0.148~0.168	0.156	0.094~0.111	0.102	34.2	18.6
	4	0.158~0.197	0.176	0.099~0.129	0.115	34.6	19.5
	6	0.157~0.222	0.184	0.093~0.159	0.123	33.2	22.0
	8	0.234~0.295	0.255	0.136~0.171	0.151	40.9	40.3
	10	0.351~0.412	0.378	0.182~0.252	0.217	42.5	49.7

**Table 9:** Influence of pure PP particles concentration on separation elements in the integrated system, of multichannel ceramic MF and pure PP particles, using air backwashing (FT 10 min, BT 10 s); paralleled with the consequences of water backwashing.

Backwashing	PP Particles (g/L)	0	5	10	20	30	40	50
Air	R_m_ × 10^−9^ (kg/m^2^/s)	1.939	0.840	0.842	0.957	0.772	0.816	0.897
	R_b_ × 10^−9^ (kg/m^2^/s)	0.051	0.029	0.002	0.034	0.004	0.010	0.025
	R_f,180_ × 10^−9^ (kg/m^2^/s)	7.045	3.252	2.586	2.460	1.932	2.175	2.110
	R_if_ × 10^−9^ (kg/m^2^/s)	3.390	1.705	1.730	1.452	1.579	1.373	1.144
	R_rf_ × 10^−9^ (kg/m^2^/s)	3.654	1.546	0.856	1.008	0.353	0.802	0.966
	J_0_ (L/m^2^/hr)	319	325	334	285	364	342	306
	J_180_ (L/m^2^/hr)	70	68	82	82	104	94	93
	J_180_/J_0_	0.220	0.211	0.246	0.287	0.286	0.275	0.304
	V_T_ (L)	1.62	1.58	1.63	1.59	2.09	2.04	2.09
Water [26]	R_m_ × 10^−9^ (kg/m^2^/s)	1.24	1.27	1.53	1.75	1.82	1.93	1.49
	R_b_ × 10^−9^ (kg/m^2^/s)	0.020	0.024	0.106	0.009	0.041	0.142	0.076
	R_f,180_ × 10^−9^ (kg/m^2^/s)	4.23	4.35	6.79	7.15	7.09	9.83	12.94
	R_if_ × 10^−9^ (kg/m^2^/s)	1.75	1.24	1.20	3.24	0.66	0.43	1.44
	R_rf_ × 10^−9^ (kg/m^2^/s)	2.486	3.110	5.587	3.910	6.423	9.403	11.50
	J_0_ (L/m^2^/hr)	503	491	388	361	342	307	405
	J_180_ (L/m^2^/hr)	116	113	75	71	71	53	44
	J_180_/J_0_	0.230	0.229	0.194	0.198	0.208	0.174	0.108
	V_T_ (L)	10.11	11.75	7.56	6.94	7.91	6.55	6.85

**Table 10:** Decay effectiveness of turbidity for influence of the PP particles concentration in the integrated system, of multichannel ceramic MF and PP particles, using air backwashing; paralleled with the consequences of water backwashing.

PP Particles (g/L)	Turbidity (NTU)				Average Decay Effectiveness (%)	
	Feed Water		Treated Water		Backwashing Media	
	Range	Average	Range	Average	Air	Water [26]
0	26.7–31.2	28.8	0.509–7.13	4.00	86.1	98.9
5	22.8–34.2	26.3	1.410–6.59	3.49	86.7	98.3
10	23.2–30.7	26.4	0.471–5.67	2.27	91.5	98.6
20	38.7–50.9	43.5	0.539–5.13	2.38	94.5	98.6
30	33.7–50.3	39.8	0.504–2.54	2.10	94.7	97.5
40	24.9–34.2	29.0	0.354–2.94	1.32	95.4	98.8
50	34.2–44.1	39.2	0.523–1.81	1.67	95.7	98.9

**Table 11:** Decay effectiveness of DOM (UV_254_ absorbance) for influence of the PP particles concentration in the integrated system, of multichannel ceramic MF and PP particles, using air backwashing; paralleled with the consequences of water backwashing.

PP Particles (g/L)	UV_254_ Absorbance (cm^−1^)				Average Decay Effectiveness (%)	
	Feed Water		Treated Water		Backwashing Media	
	Range	Average	Range	Average	Air	Water [26]
0	0.172–0.192	0.181	0.083–0.103	0.094	48.0	47.4
5	0.126–0.193	0.158	0.08–0.109	0.093	41.4	51.3
10	0.117–0.170	0.144	0.080–0.098	0.086	40.0	48.9
20	0.298–0.325	0.311	0.164–0.181	0.174	44.1	46.8
30	0.227–0.268	0.246	0.100–0.149	0.121	50.6	48.1
40	0.224–0.274	0.245	0.085–0.116	0.106	56.8	49.8
50	0.357–0.414	0.386	0.134–0.288	0.211	37.8	46.5
